# The role of mycobiota-genotype association in inflammatory bowel diseases: a narrative review

**DOI:** 10.1186/s13099-021-00426-4

**Published:** 2021-05-08

**Authors:** Elaheh Mahmoudi, Sayed-Hamidreza Mozhgani, Niusha Sharifinejad

**Affiliations:** 1 Division of Mycology, School of Medicine, Alborz University of Medical Sciences, Karaj, Iran; 2 Department of Microbiology, School of Medicine, Alborz University of Medical Sciences, Karaj, Iran; 3 Student Research Committee, Alborz University of Medical Sciences, Karaj, Iran; 4 Alborz Office of USERN, Universal Scientific Education and Research Network (USERN), Alborz University of Medical Sciences, Karaj, Iran

**Keywords:** Inflammatory bowel disease, IBD, Fungal microbiota, Intestinal mycobiota, Single nucleotide polymorphisms, SNPs

## Abstract

Inflammatory bowel disease (IBD) is a chronic inflammatory disease affecting various parts of the gastrointestinal tract. A majority of the current evidence points out the involvement of intestinal dysbiosis in the IBD pathogenesis. Recently, the association of intestinal fungal composition With IBD susceptibility and severity has been reported. These studies suggested gene polymorphisms in the front line of host defense against intestinal microorganisms are considered to play a role in IBD pathogenesis. The studies have also detected increased susceptibility to fungal infections in patients carrying IBD-related mutations. Therefore, a literature search was conducted in related databases to review articles addressing the mycobiota-genotype association in IBD.

## Inflammatory bowel disease pathogenesis

Inflammatory bowel disease (IBD) is a chronic relapsing disease affecting various parts of the gastrointestinal tract and encompasses two common disorders: Crohn’s disease (CD) and Ulcerative Colitis (UC). IBD is a worldwide issue, especially in urban and westernized countries among young individuals [1], assumed to result from an improper and continuous inflammatory response to commensal microbes in a genetically susceptible host [2]. So far, the pathogenesis of the disease is considered to be a combination of genetic predisposition and environmental factors. The majority of current evidence emphasizes the involvement of intestinal dysbiosis in IBD pathogenesis [3]. While intestinal epithelial cells (IECs) are constantly exposed to microbial components; they are regarded not only as a structural but also a functional barrier in the front line of host defense against intestinal microorganisms. The functional alteration of these cells is hypothesized to be associated with IBD [4]. Bacteria as the predominant organisms of the gastrointestinal tract gained the greatest attention in IBD microbial studies [5–7]. Nonetheless, the association of intestinal fungal composition with mucosal inflammation in both CD and UC has recently become into consideration [8–11]. Of note, increased IBD flares were associated with increased global fungal load accompanied by alteration of certain fungal species in the microbiota [12–14].

To date, numerous gene polymorphisms are found to be connected to IBD susceptibility [15] and severity; for instance, an increased colitis severity was driven by activation of Leucine-rich repeat kinase 2 (LRRK2), an important enzyme that regulates innate immunity through nuclear factor kappa B (NF-κB) signaling pathway [16]. Some articles studied the association of specific intestinal bacterial microbiota with gene polymorphisms [17, 18]. However, few have focused on the role of fungal subsets in the intestine. The purpose of this study was to discuss the association of fungal flora with IBD and review the articles connecting the gene polymorphisms with intestinal mycobiota in IBD cases.

### Anti-Saccharomyces cerevisiae antibody

The first sparks of fungi role in IBD pathogenesis flared by detecting elevated levels of anti-*Saccharomyces cerevisiae* mannan antibodies (ASCA) in the sera of IBD-affected patients since the early 90 s [19, 20]. A twin study in 2005 has detected ASCA in CD cases more frequently compared with healthy controls [21]. ASCA was also found commonly in CD patients with a positive family history of IBD [22] and even in unaffected relatives of CD patients [23]. ASCA was not only detected in answer to *Saccharomyces* antigens but also in response to *Candida albicans* or the presence of anti-β2 glycoprotein I antibodies in CD patients [24, 25]. Marrakchi et al*.* revealed a positive correlation of caspase recruitment domain-containing protein 15 (*CARD15*)/nucleotide-binding oligomerization domain-containing protein 2 (*NOD2*) gene mutation, an important intracellular pattern recognition receptor (PRR) that is expressed by dendritic cells (DCs), macrophages, and IECs [26], with ASCA expression in IBD-affected patients [27].

### IBD affecting intestinal mycobiota

In addition to animal studies, some articles are conveying the alteration of intestinal mycobiome in human subjects with IBD. Ott et al*.* first described significantly higher fungal diversity in patients with CD in comparison with healthy controls, albeit no disease-specific fungal species were present in the CD and UC group [28]. Ever since, many studies have consistently shown an elevated abundance of *Candida* sp. in IBD fecal samples [29–31]. Lewis et al*.* have reported an increased amount of *S. cerevisiae* [29], whereas Hoarau et al*.* reported a reduction in intestinal *S. cerevisiae* abundance in IBD patients [31]*.* Another study in 2009 reported a significantly elevated *C. albicans* population obtained from fecal samples of CD patients (44%) and their healthy relatives (38%) compared to healthy controls [22]. Li et al*.* assessed 19 patients with active CD and 7 healthy individuals and discovered increased fecal fungal richness and diversity in *C. albicans*, *Aspergillus clavatus*, *Cryptococcus neoformans*, and a decrease in *S. cerevisiae* in CD patients. The diversity of the fecal fungal community was also positively correlated with serum C-reactive protein level and the CD activity index [13]. Another study in 2016, revealed a significant increase in global fungal load in both inflamed and non-inflamed mucosa compared with healthy subjects (HS). However, no significant differences in fungal diversity were observed between the groups [12].

Unlike most similar articles, Chehoud et al*.* demonstrated pediatric IBD to be associated with reduced fungal diversity in the host gut microbiota. Specific *Candida* taxa were also found to have increased abundance in the IBD samples [30]. An additional study with de-novo pediatric IBD cases revealed a shift from the *Ascomycota*-predominant mycobiota in HS to a different fungal spectrum with a predominance of *Basidomycetes* in patients with de-novo IBD without the conflicting impact of antibiotics or immunosuppression [32]. Later, another study investigated the possible fungal dysbiosis index in IBD; the fecal fungal composition of 235 patients with IBD and 38 HS showed an increased *Basidiomycota*-to-*Ascomycota* ratio that was dramatically higher in patients with IBD flares compared to patients in remission and HS [8]. There was also a negative correlation between the abundance of *S. cerevisiae* and *C. albicans* in fecal samples of IBD subjects, suggesting a competitive environment between these two species in the gut [8, 33]. The study also described a complex fungal-bacterial interaction in the fecal composition of subjects [8].

As opposed to Sokol and Mukhopadhya et al., Qiu and colleagues did not detect any significant difference in the abundance of *Ascomycota*, *Basidiomycota*, and the ratio of *Ascomycota*-to-*Basidiomycota* between the HS and UC patients. However, there was a prominent variation in the abundance of *Aspergilli* between the groups [11]. A recent report studied the cultivable intestinal mycobiota presented in feces obtained from 34 pediatric CD patients, 27 pediatric UC patients, and 32 healthy children. The authors observed increased load of *S. cerevisiae* and *Candida* sp*.* in IBD patients, which was in line with previous studies. Likewise, Di Paola et al*.* concluded that the presence of *S. cerevisiae* was associated with a favorable intestinal environment for beneficial bacterial genera, such as *Faecalibacterium*; whereas the absence of normal fungal flora or presence of unusual fungal species were conjugated with the presence of potential pathogenic bacteria that might lead to IBD [34]. The latest article by Nelson et al. reported an increased abundance of *Candida* sp. and a decreased *Basidiomycota*-to-*Ascomycota* ratio, in contrast to the previous literature, in CD cases [35]*.* Of note, the discrepancies between these studies might stem from different fungal extraction methods. In this regard, we provided additional information for these studies, including the fungal extraction method and the sample source, in Table 1.

**Table 1:** IBD affecting intestinal mycobiota in IBD patients

Number of patients	Sample/method	Result	References
57 IBD patients 47 HS	Intestinal mucosa 18S rDNA-based sequencing	Significant higher fungal diversity in patients with CD in comparison with HS. No disease-specific fungal species were found in the CD and UC group	Ott et al. [28]
41 CD families composed of: 129 patients and 113 healthy relatives 14 healthy controls families composed of 76 individuals	Mouth swabs and Stool samples were processed using chromogenic medium. Mouth swabs were rubbed directly onto the medium. Stool samples were taken with an inoculation loop. Plates were incubated for 48 h at 37°℃C. The yeast species were differentiated using the specific color of the colonies. Presumptive identification of yeast species was confirmed by either Bichro-Latex-albicans for *C. albicans*, or the API 32C system for other species	Top most prevalent mycobiome in CD patients: *C.* *albicans *mouth [26 (34.7%)] stool [13( 22%)] *C. glabrate* mouth [3 (4%)], stool [1 (1.7%)] *C. tropicalis* mouth [1 (1.3%)], stool 0	Standaert-Vitse et al. [22]
19 patients with active CD 7 HS	PCR targeting fecal fungal 18S rDNA gene	Decreased *S. cerevisiae* and overrepresented *Aspergillus clavatus, C. albicans*, and *Cryptococcus neoformans* proportions were present in CD patients	Li et al. [13]
90 children with CD 26 HS children	Sequence was acquired using the Illumina HiSeq method (Illumina)	Five yeasts including, *S. cerevisiae*, *C. lusitaniae*, *Pichia jadinii* (also known as and *C. utiliz*), *C. albicans*, and *Kluyveromyces marxianus* were positively associated with CD, particularly in the setting of greater bacterial dysbiosis	Lewis et al. [29]
32 patients with IBD 90 HS	PCR primers targeting fecal fungal the ITS rDNA gene	IBD samples had significantly lower fungal diversity The most commonly observed fungi were *C. Pichia jadinii*. *C. parapsilosis*, was also more common in the pediatric IBD samples. *Cladosporium cladosporioides*, was more common in HS	Chehoud et al. [30]
9 multiplex families comprising 20 CD patients and their 28 cohabiting NCDR 4 unrelated healthy families 21 individuals with no history of CD (NCDU) living in the same geographic area	PCR primers targeting fecal fungal ITS1 rDNA gene	Increased richness in the NCDU group compared to the CD or NCDR group but no difference in the mycobiome richness of CD patients and their healthy relatives. *S. cerevisiae* tended to increase in healthy (NCDR) individuals. *C. tropicalis* was significantly abundant in CD compared to NCDR group	Hoarau et al. [31]
23 CD patients (16 in flare, 7 in remission) 10 HS	Colonic mucosa ITS2, 16S, and 18S rDNA sequencing	Global fungi load was significantly increased in both inflamed and non-inflamed mucosa compared to HS. However, no significant differences in fungal diversity between the studied groups were observed	Liguori et al. [12]
25 children with IBD 12 HS	Colonic mucosa 18S rDNA sequencing	A shift from the *Ascomycota*-predominant microbiota in HS to a different fungal spectrum with *Basidomycetes* predominance in patients with de-novo IBD	Mukhopadhya et al. [32]
235 IBD patients 38 HS	PCR primers targeting fecal fungal ITS2 rDNA gene	*S. cerevisiae* reduction in patients with IBD (vs. healthy controls) and with flare (vs. remission). Higher *Basidiomycota-to-Ascomycota* abundance ratio in patients with IBD in flare (either UC or CD) but normal ratio in remission	Sokol et al. [8]
14 UC patients15 HS	PCR primers targeting fecal fungal ITS1 and ITS2 rDNA gene	*Wickerhamomyces*, an unidentified genus of *Saccharomycetales, Aspergillus*, *Sterigmatomyces*, and *Candida* sp. showed an increasing trend in UC patients compared with HS. There was a marked difference in *Aspergillus* abundance between the groups. The proportions of *Ascomycota* and *Basidiomycota* were not significantly different between the groups	Qiu et al. [11]
93 pediatric; 34 CD, 27 UC patients, 32 HS	PCR primers targeting fecal fungal ITS1-5.8-S-ITS2 regions of rDNA gene	*S. cerevisiae* (n = 7 fecal samples) and other yeasts (*Candida* sp.; n = 5 samples) isolated from 19 CD patients *S. cerevisiae* is associated with a favorable gut environment for beneficial bacterial genera. Whilst, the absence of yeasts or the presence of other yeast species is connected with potential pathogenic bacteria	Di Paola et al. [34]
34 CD patients 47 HS without GI disease	PCR primers targeting fecal fungal ITS1 rDNA gene	*Candida* sp. was most associated with CD and *Cryptococcus sp.* with non-CD. The *Basidiomycota/Ascomycota* abundance ratio was found to be significantly lower in CD patients	Nelson et al. [35]

CD, Crohn disease; IBD, Inflammatory bowel disease; UC, Ulcerative colitis; ITS 1,2, Internal transcribed spacer 1,2; HS, Healthy subjects; *C. albicans*, *Candida albicans*; *C. Tropicalis*, *Candida tropicalis*; *C. glabrate*, *Candida glabrate*; sp., species; *C. Pichia jadinii*, *Candida Pichia jadinii*, *C. parapsilosis*, *Candida parapsilosis*; *M. restricta*, *Malassezia restricta*; *M. sympodialis*, *Malassezia sympodialis*; *S. cerevisiae*, *Saccharomyces cerevisiae*; *NCDR*, Non-CD relatives

### Innate immunity against fungi

**Fig. 1:**
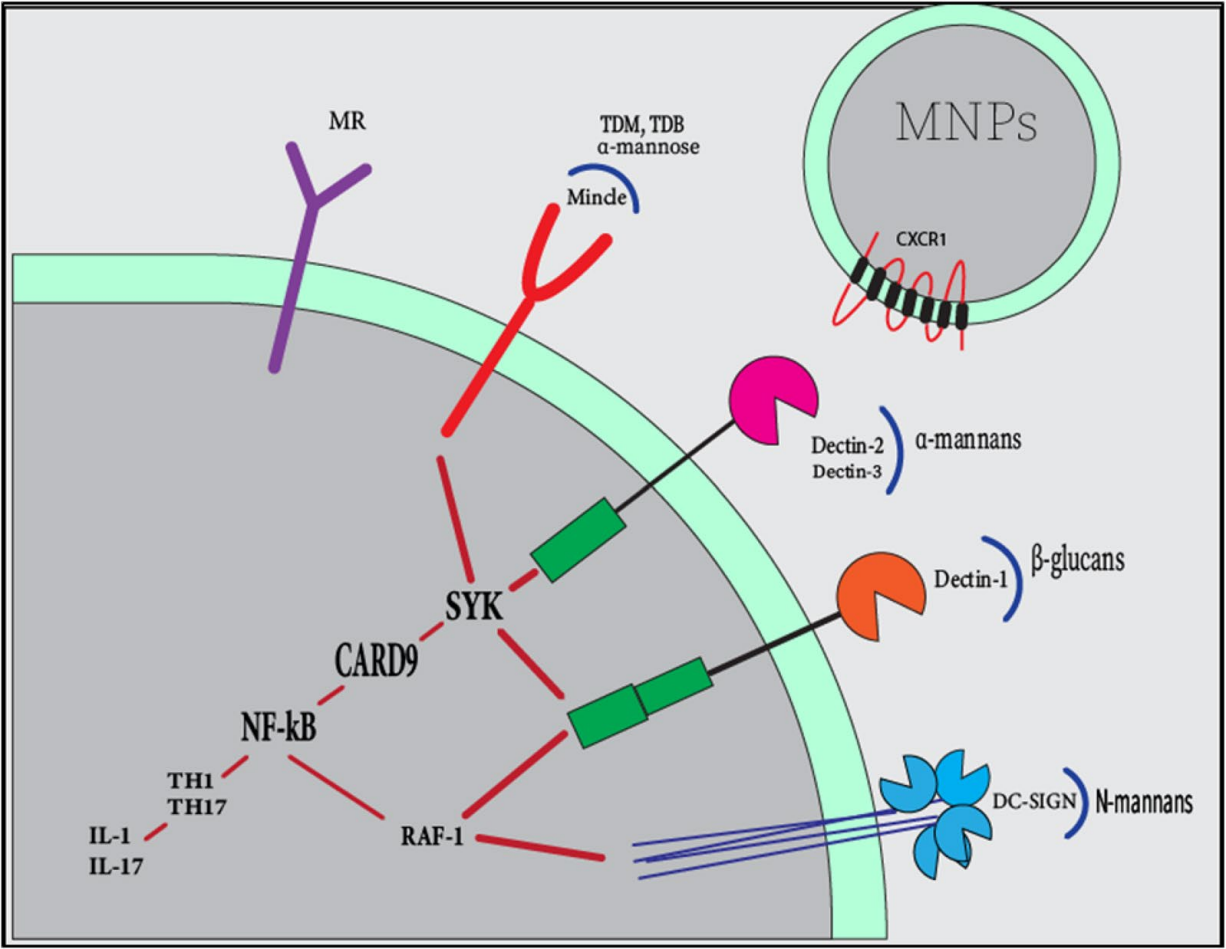
The cascade of innate immune response against intestinal fungi. Several fungal cell wall polysaccharides initiates immune responses, Dectin-1 binds β-glucans, dectin-2 binds α-mannans, and Mincle attaches the glycolipid trehalose-6,6-dimycolate (TDM), trehalose-6,6-dibehenate (TDB), and α-mannose residues. DC-SIGN binds N-linked mannans. Dectin-1, dectin-2, and mincle begin intracellular signaling through the SYK activation. RAF-1 as an SYK-independent activator of NF-κB pathway actuated by DC-SIGN and dectin-1. NF-κB pathway leads to TH1 and TH17 activations and subsequent cytokine production. CX3CR-1 is expressed by intestinal-resident mononuclear phagocytes (MNPs) and participate in fungal recognition

Several genetic polymorphisms have been detected in IBD over the years [15, 36]. The connection between various genetic polymorphisms with bacterial species in IBD patients has been widely studied [37–39]. Increased susceptibility to systemic fungal complications, such as candidemia was linked to polymorphisms of Interleukin 10 (IL-10) (rs1800896) [40], Toll-like receptors 1 (TLR-1) (rs5743611, rs4833095, rs5743618) [41], Toll-like receptors 2 (TLR-2) [42], caspase recruitment domain-containing protein 9 (CARD9) (G72S,R373P,Q295X) [43, 44], Toll-like receptors 4 (TLR-4) (rs4986790,rs4986791) [45], and Dectin-1 [46] since 2006. Chronic mucocutaneous candidiasis is also related to Toll-like receptors 3 (TLR3) (rs3775291) and Dectin-1 mutations [46–48]. Gene polymorphisms targeting innate immunity may play an important role in IBD. Although studies aiming at the role of intestinal fungi pathogenesis in IBD are scarce, studies focusing on innate immune pathways against intestinal bacteria and their inflammatory consequences have successfully revealed important roles for innate immunity in IBD. Similarly, fungal recognition in the gut may be also regulated by innate immunity [49]. Four main types of innate immune receptors that can recognize fungi through fungal Pathogen-associated molecular patterns (PAMPs) are TLRs, C-type lectin receptors (CLRs), NOD-like receptors (NLRs), and galectin 3 on antigen-presenting cells [50]. The most studied class are the CLRs which include Dectin-1, Dectin-2, Dendritic cell-specific intercellular adhesion molecule-3-grabbing non-integrin receptor (DC-SIGN), Macrophage inducible Ca^2+^-dependent lectin receptor (MINCLE), and the Mannose Receptor (MR). Additionally, some CLRs can interact with TLRs to recognize fungi [51]. The β-glucan is the main PAMP that can be recognized by Dectin-1, although Dectin-1 can also recognize unidentified bacterial and endogenous ligands [52]. Dectin-2 has been recently shown to be the functional receptor for α-mannans and to be implicated in anti-bacterial immunity [53]. The α-mannose is also strongly suggested to be Mincle’s ligand, which has been implied in anti-mycobacterial immune activity [54]. The fractalkine receptor (CX3CR-1) expressed by intestinal-resident mononuclear phagocytes (MNPs), were also characterized to have a role in initiating immune responses against fungi [55]. Through fungal recognition, these pathways initiate the inflammatory cascade by predominantly driving the immune responses through spleen tyrosine kinase (SYK)-dependent, SYK-independent, and eventually NF-κB signaling pathway towards T helper 1 (TH1) and/or T helper 17 (TH17) immunophenotypes [56]. The brief signaling cascade leading to intestinal inflammation is available in Fig. 1.

### Intestinal mycobiota-genotype association

As Table 2 represents, here, we concentrated on articles reporting the mutations of innate immunity components and resulted in the gut mycobiome alteration. In a recent article, Limon et al*.* expressed that colonization of the colonic mucosa with *Malassezia restricta*, a commensal fungus typically found on the skin, might increase IBD severity in patients with *CARD9*^*S12N*^ risk allele. They found out that the *CARD9*^*S12N*^ variant induces a potent pro-inflammatory cytokine response against *M. restricta* in IBD [57]. By examining the SYK-CARD9 signaling axis and gut fungi, Malik et al. also demonstrated the decreased occurrence of *Ascomycetes* along with elevation of *Saccharomycetes* in *Card9*^*−/−*^ mice. They implied that a normal inflammasome assembly in an unperturbed *SYK-CARD9* signaling axis led to protection against colitis and colon cancer and also promoted T cell-mediated anti-tumorigenic responses; thereby indicating that a healthy gut mycobiota could prevent the development of IBD [58]. According to Lamas et al., the fungal microbiota of wild type and *Card9*^*−/−*^ mice with induced-colitis mainly were members of the *Ascomycota*, *Basidiomycota*, and *Zygomycota* phyla. However, there were different measurements at the days 0, 7, and 12, and both groups reached a peak at day 7 that was higher in *Card9*^*−/−*^ mice. On day 7, *Card9*^*−/−*^ mice showed decreased fecal *Ascomycota*, increased fecal *Basidiomycota*, and *Zygomycota* communities [59].

**Table 2:** Intestinal mycobiota-genotype association in IBD

Animal/ human sample	Fungal extraction	Mycobiota-genotype	References
CD patients Mucosal-tissue	The ITS1 rDNA sequencing	*M. restricta (CARD9 S12N alleles)*	Limon et al. [57]
Card9^−/−^ mice feces	18S ITS rDNA sequencing	Decreased *Ascomycota*, elevation of *Saccharomycetes (CARD9)*	Malik et al. [58]
Card9^−/−^ mice feces	ITS2 rDNA sequencing	*Ascomycota, Basidiomycota, and Zygomycota (CARD9)*	Lamas et al. [59]
CX3CR1^−/−^ mice, CD patients	enzyme-linked immunosorbent assay (ELISA)	Decreased antibody production against *Candida* sp. *(CX3CR-1) [CX3CR-1 T280M (rs3732378)]*	Leonardi et al. [62]
IBD patients Fecal samples	ITS2 rDNA sequencing	Positive correlation: *M. sympodialis [Dectin-1 (rs2078178, rs3901533)],[TLR1 (rs4833095, rs5743618)],[Mincle (rs10841845)]* *S. cerevisiae [CARD9 (rs10781499)], [TLR3 (rs3775291)]* *Ascomycota [DC-SIGN (rs2287886)], [TLR1 (rs5743611)]* *Basidiomycota [TLR1 (rs5743611)]* Negative correlation: *M. sympodialis [Dectin-1 (rs2078178, ‘T’allele 12)]* *S. cerevisiae [CARD9 (rs10781499, ‘A’ allele 21)]*	Sokol et al. [8]
Clec4d^−/−^ mice feces	18S rDNA sequencing	*C. tropicalis (CLEC4D)*	Wang et al. [64]
Clec7^−/−^ mice feces	ITS1-2 rDNA sequencing	Increased *Candida* and *Trichosporon* sp. Decreased nonpathogenic *Saccharomyces* sp.	Iliev et al. [66]
CD patients Fecal sample	ITS1 rDNA sequencing	No differences were evident with *NOD2* variances	Nelson et al. [35]

ITS 1,2, Internal transcribed spacer 1, 2; CARD9; CARD9, Caspase recruitment domain-containing protein 9; TLR3, Toll-like receptors 3; TLR1, Toll-like receptors 1; CLEC4D, C-Type Lectin domain containing 4D; CLEC7A, C-Type Lectin domain containing 7A; DC-SIGN, Dendritic cell-specific intercellular adhesion molecule-3-grabbing non-integrin receptor; MINCLE, Macrophage inducible Ca^2+^-dependent lectin receptor; NOD2, Oligomerization domain-containing protein 2; *M. restricta, Malassezia restricta; M. sympodialis, Malassezia sympodialis; S. cerevisiae, Saccharomyces cerevisiae; C. tropicalis, Candida tropicalis*; sp., species

*CX3CR-1 T280M (rs3732378)* is a common polymorphism that has been previously detected in extra-intestinal inflammatory diseases [60, 61]. In 2018, Leonardi et al. described that CX3CR1 + MNPs not only modifies adaptive immune responses to intestinal fungi and controls the mycobiota during experimental colitis in animal models (without changing bacterial communities), but is also connected with a decrease in antifungal antibody responses in CD patients. They concluded that intestinal mycobiota and *CX3CR1*-dependent immune responses might provoke extra-intestinal manifestations of inflammatory diseases [62]. Elevated antifungal antibodies detected in patients with alcoholic liver disease, Graves’ disease, spondyloarthritis, and systemic lupus erythematous corroborate this hypothesis [63]. Finally, the article provided evidence for CX3CR1 + MNPs as a mediator between gut mycobiome and both local and systemic immunity [55].

A previous study was conducted by Sokol et al. to examine the correlation between host genotype and fungal microbiota in IBD patients. The ten most significant connections between IBD-associated fungi taxa and single-nucleotide polymorphisms (SNPs) were as follows: *Malassezia sympodialis* association with Dectin-1 (*rs2078178, rs3901533*), TLR1 (*rs4833095, rs5743618*), and Mincle (*rs10841845*); *S. cerevisiae* with CARD9 (*rs10781499*) and TLR3 (*rs3775291*); *Ascomycota* with DC-SIGN (*rs2287886*) and TLR1 (*rs5743611*); and *Basidiomycota* with TLR1 (*rs5743611*). They also provided evidence supporting the negative correlation of *M. sympodialis* fecal abundance with Dectin-1 SNP (*rs2078178, ‘T’allele 12*) in medically refractory UC; *M. sympodialis* was also decreased during the IBD flares in patients. Moreover, the IBD-associated CARD9 variation (*rs10781499, ‘A’ allele 21*) was inversely correlated with the fecal abundance of *S. cerevisiae*. Lastly, they reported a decrease in fungal biodiversity only in UC and CD patients without ileal involvement [8].

Wang et al*.* described the role of Dectin-3 (a family member of CLRs) in recognizing *Candida. tropicalis* in experimental-colitis pathogenesis for the first time. They observed that *C. tropical* increased the disease burden in *Clec4d*^*−/−*^ mice during the induced colitis. Since the C-Type Lectin domain containing 4D (*CLEC4D*) is the encoding gene for Dectin-3, *Clec4d*^*−/−*^ mice were more susceptible to induced colitis due to the activation of the NF-κB signaling pathway64.

The impact of NOD2 variants on the intestinal bacterial community in CD patients has previously been described [65]. Thus, Nelson et al*. *investigated the presence of NOD2 polymorphisms in CD patients and its relation with fecal fungal diversity but did not find any significant correlation between NOD2 variants and specific intestinal fungi community [35].

Dectin-1 is the most important fungal PRR expressed by innate immune cells, such as macrophages, dendritic cells, and neutrophils. C-Type Lectin domain containing 7A (*CLEC7A*) is the gene that encodes Dectin-1. *Clec7*^*−/−*^ mice with induced colitis had increased proportions of opportunistic pathogenic fungi including *Candida* sp*.* and *Trichosporon* sp*.* along with a decreased frequency of nonpathogenic *Saccharomyces*. Iliev et al*.* identified a significant association between *CLEC7A* SNP (*rs2078178*) and patients suffering from medically refractory UC and delineated the role of Dectin-1 as a fungal receptor during severe forms of colitis [66]. Other gene polymorphisms were also described to influence Dectin-1-associated immunity in IBD [16, 67]. Among these genes, *LRRK2* has also been described as the familial Parkinson’s disease genetic risk factor. Multiple variations in *LRRK2* comprising *N2081D*, *rs11175593 LRRK2/MUC19*, and *rs11564258 LRRK2/MUC19* were associated with IBD as well [68]. Takagawa et al*.* suggested an increase in severity of colitis, mediated by increased Dectin-1–induced immunity, in (*rs11564258*) *LRRK2/MUC19* polymorphism carriers [16]. Noteworthily, this variance (*rs11564258*) had the second-highest odds ratio in IBD patients of the European population [69]. Further studies are required to identify the intestinal mycobiota in the patients carrying this mutation.

## Conclusion

In summary, the role of intestinal fungal mycobiota in IBD pathogenesis and severity index have been quite underrated. This review emphasizes that a majority of IBD-affected patients had increased diversity and richness of intestinal mycobiome, higher abundance of *C. albicans* and *Basidiomycota*-to-*Ascomycota* ratio, and a decreased proportion of *S. cerevisiae* despite a few contradictory results in other studies.

It is widely known that innate immunity takes part in intestinal fungal recognition and mutations in innate immunity mediators are linked to IBD pathogenesis. Even so, few articles aimed to examine the connection between gene polymorphisms and intestinal fungal dysbiosis in IBD.

Although DSS-induced colitis is a well-established experimental murine model with much resemblance to human IBD [70], we were able to find only three non-murine studies containing mycobiota-genotype data related to IBD patients. Additional evidence is needed to determine whether different gene polymorphisms can alter intestinal mycobiome or whether this information would be of use in providing novel insight into IBD pathogenesis. Therefore, our purpose was to highlight the importance of the matter and draw attention to this underappreciated aspect of IBD-associated research.

## Abbreviations

IBD: Inflammatory bowel disease
CD: Crohn’s disease
UC: Ulcerative Colitis
IECs: Intestinal epithelial cells
LRRK2: Leucine-rich repeat kinase 2
ASCA: Anti-Saccharomyces cerevisiae antibody
CARD15: Caspase recruitment domain-containing protein 15
NOD2: Nucleotide-binding oligomerization domain-containing protein 2
PRR: Pattern recognition receptor
DCs: Dendritic cells
IL-10: Interleukin 10
TLR-1: Toll-like receptors 1
TLR-2: Toll-like receptors 2
CARD9: Caspase recruitment domain-containing protein 9
TLR-4: Toll-like receptors 4
PAMPs: Pathogen-associated molecular patterns
CLRs: C-type lectin receptors
NLRs: NOD-like receptors
DC-SIGN: Dendritic cell-specific intercellular adhesion molecule-3-grabbing non-integrin receptor
MINCLE: Macrophage inducible Ca^2^^+^-dependent lectin receptor
MR: Mannose Receptor
MNPs: Mononuclear phagocytes
SYK: Spleen tyrosine kinase
SNPs: Single-nucleotide polymorphisms
TLR3: Toll-like receptors 3
CLEC4D: C-Type Lectin domain containing 4D
CLEC7A: C-Type Lectin domain containing 7A
TH1: T helper 1
TH17: T helper 17
IL-1: Interleukin 1
IL-17: Interleukin 17
